# Motor impulsivity but not risk-related impulsive choice is associated to drug intake and drug-primed relapse

**DOI:** 10.3389/fnbeh.2023.1200392

**Published:** 2023-06-02

**Authors:** Chloé Arrondeau, Ginna Urueña-Méndez, Lidia Bellés, Florian Marchessaux, Raphaël Goutaudier, Nathalie Ginovart

**Affiliations:** ^1^Department of Psychiatry, Faculty of Medicine, University of Geneva, Geneva, Switzerland; ^2^Department of Basic Neurosciences, Faculty of Medicine, University of Geneva, Geneva, Switzerland

**Keywords:** motor impulsivity, risk-related impulsive choice, drug abuse, cocaine, aripiprazole, dopamine, self-administration (SA)

## Abstract

**Introduction:**

Motor impulsivity and risk-related impulsive choice have been proposed as vulnerability factors for drug abuse, due to their high prevalence in drug abusers. However, how these two facets of impulsivity are associated to drug abuse remains unclear. Here, we investigated the predictive value of both motor impulsivity and risk-related impulsive choice on characteristics of drug abuse including initiation and maintenance of drug use, motivation for the drug, extinction of drug-seeking behavior following drug discontinuation and, finally, propensity to relapse.

**Methods:**

We used the Roman High- (RHA) and Low- Avoidance (RLA) rat lines, which display innate phenotypical differences in motor impulsivity, risk-related impulsive choice, and propensity to self-administer drugs. Individual levels of motor impulsivity and risk-related impulsive choice were measured using the rat Gambling task. Then, rats were allowed to self-administer cocaine (0.3 mg/kg/infusion; 14 days) to evaluate acquisition and maintenance of cocaine self-administration, after which motivation for cocaine was assessed using a progressive ratio schedule of reinforcement. Subsequently, rats were tested for their resistance to extinction, followed by cue-induced and drug-primed reinstatement sessions to evaluate relapse. Finally, we evaluated the effect of the dopamine stabilizer aripiprazole on reinstatement of drug-seeking behaviors.

**Results:**

We found that motor impulsivity and risk-related impulsive choice were positively correlated at baseline. Furthermore, innate high levels of motor impulsivity were associated with higher drug use and increased vulnerability to cocaine-primed reinstatement of drug-seeking. However, no relationships were observed between motor impulsivity and the motivation for the drug, extinction or cue-induced reinstatement of drug-seeking. High levels of risk-related impulsive choice were not associated to any aspects of drug abuse measured in our study. Additionally, aripiprazole similarly blocked cocaine-primed reinstatement of drug-seeking in both high- and low-impulsive animals, suggesting that aripiprazole acts as a D_2/3_R antagonist to prevent relapse independently of the levels of impulsivity and propensity to self-administer drugs.

**Discussion:**

Altogether, our study highlights motor impulsivity as an important predictive factor for drug abuse and drug-primed relapse. On the other hand, the involvement of risk-related impulsive choice as a risk factor for drug abuse appears to be limited.

## 1. Introduction

Substance use disorder (SUD) is characterized by a loss of control over drug use, a high motivation for the drug, drug consumption in spite of negative consequences (i.e., compulsivity), and vulnerability to relapse despite abstinence (American Psychiatric Association, 2013). One of the major challenges in the field is to identify the vulnerability factors associated with SUD since only a portion of users, between 4 and 30%, progress toward the disorder (Anthony and Helzer, 2002). Impulsivity has long been suggested as a risk factor for SUD (review in Weafer et al., 2014). This multifaceted personality trait (Evenden, 1999), includes motor impulsivity, defined as the inability to withhold an action, and choice impulsivity, which can itself be divided into risk- and delay-related impulsive choice (Winstanley et al., 2006; Jentsch et al., 2014). High levels of both motor impulsivity and risk-related impulsive choice have been reported in patients with SUD (Bechara et al., 2002; Verdejo-García et al., 2007; Ersche et al., 2010; Urcelay and Dalley, 2011; Jentsch et al., 2014; Reddy et al., 2014). However, it is difficult to disentangle in humans whether these high impulsive behaviors precede or are consequences of drug abuse.

Mixed preclinical results exist on the predictive value of motor impulsivity on different aspects of drug abuse. Although motor impulsivity has been linked to compulsive drug use (Belin et al., 2008) and greater resistance to extinction of drug-seeking (Diergaarde et al., 2008), previous studies have reported either increased levels of drug taking (Dalley et al., 2007), or no difference in drug taking (Belin et al., 2008; Caprioli et al., 2013; Abbott et al., 2022) in high compared to low impulsive animals. Further studies are therefore necessary to disentangle the predictive value of motor impulsivity on vulnerability to drug abuse. On the other hand, only a limited number of studies have evaluated the predictive value of decision-making on different aspects of substance abuse. It has been suggested that risk-related impulsive choice predicts drug-intake in adolescent but not in adult animals (Mitchell et al., 2014), and that, in adults, risky decision-making might be associated to incubation of craving but not to drug taking (Ferland and Winstanley, 2017). However, further studies are still required to investigate whether risk-related impulsive choice predicts other hallmarks of addiction, such as motivation for drug use or vulnerability to relapse following drug discontinuation.

The Roman High- (RHA) and Roman Low Avoidance (RLA) rat sublines were originally derived from Wistar rats based on their avoidance behavior in the shuttle box (Bignami, 1965). In addition to this initial phenotype difference, RHA and RLA rats differ in other behavioral characteristics. Compared to RLA rats, RHAs show higher levels of novelty seeking (Tournier et al., 2013; Bellés et al., 2021a), motor impulsivity (Moreno et al., 2010; Bellés et al., 2023) and risk-related impulsive choice (Bellés et al., 2023), but lower levels of anxiety-related behaviors (review in Giorgi et al., 2019). As novelty seeking, impulsive behaviors and anxiety have been reported as risk-factors for susceptibility to drug abuse (review in Belin et al., 2016), the RHA and RLA rat sublines provide a valuable model for studying the neurobiological processes underlying vulnerability to drug abuse. Interestingly, RHA rats also display higher levels of cocaine taking during self-administration (SA) paradigm (Fattore et al., 2009; Dimiziani et al., 2019). Noticeably, and consistent with previous results obtained in Lister Hooded rats (Dalley et al., 2007), high motor impulsivity in RHA rats has been associated with a reduced density of dopamine (DA) D_2/3_ receptors (D_2/3_R) in the striatum (Bellés et al., 2021b), but also to a higher magnitude of striatal amphetamine-induced DA release when compared to low impulsive RLA rats (Tournier et al., 2013), thus emphasizing a role of alterations in central dopaminergic functioning in motor impulsivity (reviewed in Dalley and Ersche, 2019). Besides, and although scarce, support for a DA involvement in risk-related impulsive choice comes from preclinical studies showing that D_2/3_R blockade improves risk-related impulsive choice (Zeeb et al., 2009), whereas D_2/3_R stimulation worsens it (Barrus and Winstanley, 2016; Freels et al., 2020; Bellés et al., 2023). Collectively, these data suggest that innately low levels of striatal D_2/3_R combined with an elevated presynaptic DA tone result in a specific pattern of DA signaling that may promote motor impulsivity and risk-related impulsive choice in RHA rats. Interestingly, DA stabilizers, such as aripiprazole, have been studied as potential candidates for treating dependence to different types of drugs (reviewed in Brunetti et al., 2012). Aripiprazole is a D_2/3_R partial agonist whose properties are dependent on the endogenous levels of DA. Indeed, aripiprazole displays D_2/3_R antagonistic properties in hyperdopaminergic states, and agonistic properties in hypodopaminergic states (for review, see de Bartolomeis et al., 2015). In rodents, stabilizing DA transmission with a systemic injection of aripiprazole has been shown to attenuate motor impulsivity (Besson et al., 2010), and to decrease cocaine- and cue-induced reinstatement of drug-seeking behavior (Feltenstein et al., 2007, 2009). As RHA rats exhibit a hyper-reactive striatal dopamine system compared to RLA rats (Tournier et al., 2013; Bellés et al., 2021b), we sought to determine whether a stabilization of DA neurotransmission with aripiprazole had a differential effect on drug-seeking behavior in RHA and RLA rats.

In this study, we used a cocaine SA paradigm to evaluate the predictive value of both motor impulsivity and risk-related impulsive choice on the initiation and maintenance of drug use, motivation for the drug using a progressive ratio (PR) schedule of SA, extinction of drug-seeking behavior and then cue-induced or drug-primed reinstatement of drug-seeking. Additionally, we tested the effect of aripiprazole on reinstatement of drug-seeking in both lines.

## 2. Materials and methods

### 2.1. Animals

A total of 15 RHA and 15 RLA adult male rats, aged 2 months and weighing 250–300 g at the beginning of experiments, were used from our colony at the University of Geneva. Rats were housed by two or three in a room maintained on a 12:12 h light-dark cycle (lights on at 7:00 a.m.). Access to food was restricted to 5 g per 100 g of body weight per day in order to maintain animals at 85–90% of their free-feeding weight. Water was available *ad libitum*. Behavioral sessions were conducted daily, between 8 and 17 h, 5 days per week. All animal experiments were performed in accordance with the Swiss Federal Law on animal care and to the European Union Directive (2010/63/EU), and were authorized by the Cantonal Veterinary Office of Geneva.

### 2.2. Drugs

Cocaine hydrochloride (Pharmacy of the Geneva University Hospitals) was dissolved in saline. Aripiprazole (Tocris, Bristol, UK) was suspended in 1 mL distilled water and 5% TWEEN. For perioperative care, amikacin (Bristol-Myers Squibb, Cham, Switzerland), cefazolin (Labatec Pharma, Meyrin, Switzerland) and buprenorphine (Reckitt Benckiser, Wallisellen, Switzerland) were dissolved in saline.

### 2.3. Behavioral tasks

#### 2.3.1. Rat gambling task (rGT)

The rGT was performed as previously described (Zeeb et al., 2009). Briefly, sessions were performed in operant conditioning chambers (Med Associates Inc., St Albans, VT, USA) individually enclosed in a sound-attenuating cubicle. Each chamber was equipped with a house-light, 5 holes positioned 2.5 cm above the floor and equipped with a cue light and an infrared head entry detector. The middle hole was not used. Nose-poke responses in the holes were detected with an infrared detector. Rodent Dustless Precision Pellets^®^ (45 mg Noyes dustless pellets, TestDiet^®^, St Louis, MO, USA) were delivered at the opposite wall via a dispenser into a food receptacle. Chambers were controlled with the MedPC IV software (Med Associates Inc., St Albans, VT, USA). Rats were first habituated to the operant boxes for 2 sessions of 30 min before being trained to nose-poke in an illuminated hole to receive a food reward (pellet). The order of the holes illuminated was pseudo-randomized. They were then trained in 7 forced-choice sessions, where only one option per trial was presented. Finally, rats were tested for 25 free-choice sessions, lasting 30 min each. A trial started after a nose-poke into the illuminated food receptacle. All the lights were turned off for an inter-trial interval (ITI) of 5 s, during which rats had to abstain from responding into the choice holes. Nose-poking during the ITI was registered as a premature response, which resulted in the illumination of the box for 5 s. The light in the food receptacle was turned on again for the initiation of the next trial. After the ITI, the four holes were illuminated for 10 s of limited hold, during which rats could nose-poke in any hole. Each hole was associated with different probabilities of reward, counterbalanced between rats: P1 (0.9), P2 (0.8), P3 (0.5), and P4 (0.4) of receiving different magnitudes of reward (1, 2, 3, or 4 pellets, respectively) and different durations of time-out (TO) punishment (5, 10, 30, 40 s, respectively). If the trial was rewarded, the corresponding number of pellets were delivered in the food tray. If the trial was punished, the light of the hole blinked at 0.5 Hz for the TO duration. An absence of response within 10 s was recorded as an omission and the trial reinitialized. P1 and P2, considered as “optimal choices,” were the choices leading to the maximum pellets earned at the end of a session. Conversely, P3 and P4, considered as “non-optimal choices” lead to longer TO punishments and less pellets earned over the session. The percentage of choices for an option was calculated as the number of choices for this option/total number of choices × 100. Risk-related impulsive choice was evaluated with the choice score (% optimal choices -% non-optimal choices). Baseline measures were established across 3 consecutive sessions. To assess baseline stability, a mixed factorial ANOVA was performed with line (i.e., RHA or RLA) as between-subjects factor, and the choice score of the 3 selected sessions as a within-subjects factor. Baseline sessions were considered stable if no main effect of session, and no interaction line × sessions were observed. Then, the choice score and the percentage of premature responses were averaged over the 3 baseline sessions. Rats with a choice score >50 were considered as having an “optimal” decision-making profile, whereas rats with a choice score <50 were considered as having a “non-optimal” profile. Motor impulsivity was assessed by the percentage of premature responses (number of premature responses/total number of trials × 100).

#### 2.3.2. Cocaine self-administration

Following the rGT, rats were anesthetized under 2% isoflurane anesthesia to insert a catheter in the left jugular vein. The distal part of the jugular catheter exited in the midscapular region and was connected to a vascular access button (VAB; Instech Laboratories, Plymouth Meeting, PA, USA). The VAB provided aseptic intravenous access, and was sealed with an aluminum cover cap (Instech Laboratories, Plymouth Meeting, PA, USA) to protect the catheter base from cage mates. After 7 days of recovery, rats were trained to self-administer cocaine. Cocaine SA was performed in operant conditioning chambers (Med Associates Inc., St Albans, VT, USA) equipped with a house-light, 2 nose-poke holes, one active and one inactive, positioned 2.5 cm above the floor, each fitted with a cue light and an infrared head entry detector. Drug infusion was delivered through a polyurethane tube protected by a metal spring and attached to a counterbalanced swivel, connected to an infusion pump. Rats were trained to self-administer cocaine under a fixed ratio 1 (FR1) schedule of reinforcement. Two-hours daily sessions started with rats receiving a priming infusion of 0.3 mg/kg cocaine, a dose that supports cocaine SA in both RHA and RLA rats (Dimiziani et al., 2019). Nose-poking into the active hole resulted in the infusion of 0.06–0.1 ml of cocaine over 2.5–4 s associated with the illumination of the cue light for the duration of infusion. Infusions were followed by a 20 s time-out, signaled by all lights being turned off, and during which nose-poking in any of the holes was recorded but without consequences. Sessions were terminated after 60 infusions or after 120 min had elapsed. Rats were tested for 15 consecutive sessions. Cocaine SA was acquired once the rats reach ≥70% discrimination between the active over the inactive hole, with ≥15 active nose-pokes.

#### 2.3.3. Progressive ratio

Motivation for cocaine intake was assessed using a PR schedule of reinforcement during which the number of nose-pokes required to obtain a single infusion increase exponentially within the sessions (1,1,2,4,6,9,12,15,20,25,32,40,50,62,77,95,118,145,178,219, 268,328,402,492,603,737,901,1102,1347,1647). The breaking point (BP) was defined as the maximum number of nose-pokes reached to obtain a single infusion (Roberts et al., 1989). Sessions ended after 3 h or after 45 min of inactivity, whichever came first. Rats were tested for 3 days, and the average BP and total active nose-pokes of the sessions were used for the analyses. One RHA rat and one RLA rat from the optimal group were excluded because of catheter patency loss.

#### 2.3.4. Extinction and reinstatement

Rats were first tested in a FR1 schedule for 2 days, to establish baseline levels of cocaine infusions. Rats were then tested in 14 daily extinction sessions of 2 h, during which nose-poking was not rewarded. Drug-seeking behavior was considered extinguished when rats did less than 20 nose-pokes in the active and inactive hole. One day after the last extinction session, rats received cue- or drug-induced reinstatement sessions, during which the house light was turned on and the nose-pokes in active and inactive holes were recorded but without consequences. We evaluated the effect of pre-treatment with cocaine (5 mg/kg, i.p., immediately prior to the session), the DA D_2/3_R partial agonist aripiprazole (1 mg/kg, i.p., at 20 min prior to the session), and pre-treatment with both cocaine and aripiprazole on drug seeking. For cue-induced reinstatement, nose-poking in the active hole resulted in the presentation of the same drug-paired cue-light presented during SA. Each reinstatement session lasted 2 h and was preceded by a session in vehicle conditions. The order of reinstatement sessions was counterbalanced across rats.

### 2.4. Statistical analyses

Normality of the data was assessed using a Shapiro-Wilk test. Data were analyzed according to the line (i.e., RHA vs. RLA rats) or the risk-related impulsive choice profile (i.e., optimal vs. non-optimal rats) of the animals. Between-group differences in the% premature responses and choice scores in the rGT were tested using unpaired Student’s *t*-test if data were normally distributed, or Mann-Whitney *U*-tests for non-normal data. Acquisition of cocaine SA was evaluated as the percentage of rats reaching the criterion of acquisition. Between-line or between-profile differences were evaluated using Log-rank Mantel-cox survival test. The number of infusions and the number of total active responses during cocaine SA were averaged for the 3 last sessions and data were analyzed using unpaired *t*-test or Mann-Whitney *U*-test, depending on the normality of the data. Correlations between the percentage of premature responses or choice scores, and the number of days required to acquire cocaine SA, the average number of infusions over the 3 last sessions, or the average number of total active responses over the 3 last sessions, were performed using Pearson’s correlation coefficient for normally distributed data and Spearman’s rank correlation coefficient for non-normally distributed data. Prior to ANOVA analyses, homogeneity of variances was verified with Levene’s test. Sphericity was assessed using Mauchly’s test, and the degrees of freedom were corrected using Greenhouse-Geisser correction if the sphericity assumption was violated. The number of infusions earned and total number of active and inactive responses performed during cocaine SA and during extinction sessions were analyzed using a mixed factorial ANOVA varying line or profile as between-subject factor, and sessions as within-subject factor. Outliers were identified using Grubb’s test and data were then winsorized. For the analyses of reinstatement, non-normal data were normalized using square root transformation. The total number of nose-pokes during each reinstatement session was analyzed using a two-way mixed factorial ANOVA, with line or profile as between-subject factor, and with treatments or cue and their corresponding vehicle session as within-subject factor. *Post hoc* comparisons were done using a Bonferroni’s test with multiple comparison correction. Data were considered significant when *p* < 0.05 and are presented as mean ± SEM. Statistical tests were performed with SPSS (IBM SPSS Statistics 27) and the figures with Graphpad Prism (Graphpad Software 9.0.2, San Diego, CA, USA).

## 3. Results

### 3.1. Rat gambling task

**FIGURE 1:**
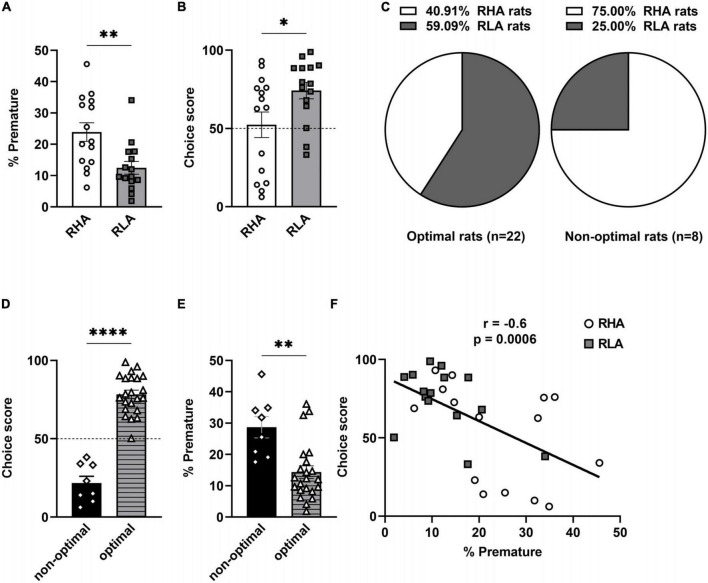
Baseline performances in the rGT. Differences between RHA (*n* = 15) and RLA rats (*n* = 15) in the percentage of premature responses **(A)** and choice scores **(B)** averaged across 3 consecutive rGT sessions at baseline. **(C)** Proportions of RHA and RLA rats in the optimal (*n* = 22) and the non-optimal group (*n* = 8). Differences in choice scores **(D)** and premature responses **(E)** between optimal and non-optimal rats. **(F)** Negative correlation between choice scores and percentages of premature responding in the rGT. Data are represented as mean ± SEM; *p < 0.05, **p < 0.01, ****p < 0.0001.

Consistent with previous studies from our group (Bellés et al., 2023), RHA rats showed a higher percentage of premature responses (*t* = 3.17, df = 28, *p* = 0.004; Figure 1A) and a lower choice score (*U* = 161, *p* = 0.045; Figure 1B) in the rGT, indicating higher levels of both motor impulsivity and risk-related impulsive choice in RHAs when compared to RLA rats. In addition, RHA rats omitted less trials than RLA rats (RHA: 5.4 ± 3.2%; RLA: 14.3 ± 6.9%; *U* = 28, *p* = 0.0002). When rats were separated according to their decision-making profile, irrespective of their line (Figure 1C), the optimal group was composed of a majority of RLA rats (9 RHA and 13 RLA rats), whereas the non-optimal group was in majority composed of RHA rats (6 RHA and 2 RLA rats). The non-optimal group exhibited a mean choice score of 21.65 (range: 6–38), whereas the optimal group had a significantly higher mean choice score of 78.24 (range: 51–99; *t* = 10.8, df = 28, *p* < 0.0001; Figure 1D). Furthermore, non-optimal and optimal animals displayed similar levels of omissions (non-optimal: 7.5 ± 5.2%; optimal: 10.7 ± 3.2%; *t* = 1.10, df = 28, *p* = 0.28). Non-optimal decision-makers displayed higher levels of premature responding (*U* = 21, *p* = 0.001; Figure 1E), indicating a higher motor impulsivity compared to the optimal group. In addition, a negative correlation was found between the percentage of premature responses and the choice score (*r* = −0.6; *p* = 0.0006; Figure 1F), indicating that higher motor impulsivity is associated to riskier decision-making.

### 3.2. Acquisition and maintenance of cocaine SA

**FIGURE 2:**
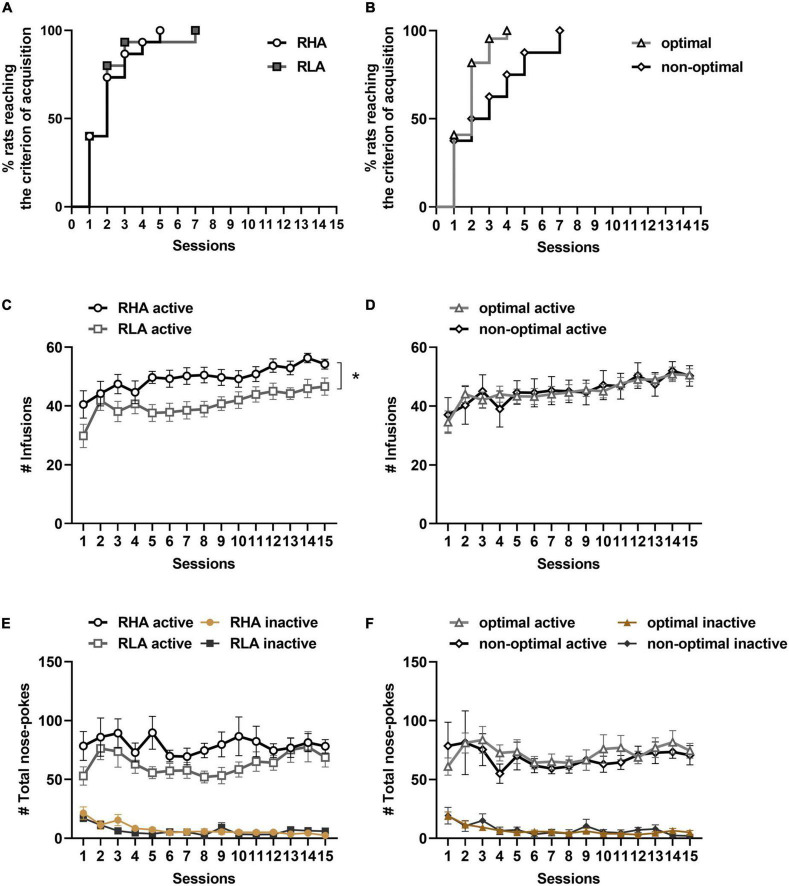
Behavioral differences in the acquisition and maintenance of cocaine SA. Cumulative proportions of rats reaching the SA acquisition criterion, comparing RHA (*n* = 15) and RLA (*n* = 15) rats **(A)**, and optimal (*n* = 22) and non-optimal (*n* = 8) rats **(B)**. Number of cocaine infusions between RHA and RLA rats **(C)**, and between optimal and non-optimal rats **(D)** over the 15 days of cocaine SA. Total number of active and inactive responses over the 15 sessions of cocaine SA in RHA vs. RLA rats **(E)**, and in optimal vs. non-optimal rats **(F)**. Data are represented as mean ± SEM. *p < 0.05.

Rats were trained to cocaine SA under a FR1 schedule of reinforcement. No differences in the rate of cocaine SA acquisition were found between RHA and RLA rats (χ^2^ = 0.0006, df = 1, *p* = 0.98; Figure 2A), or between optimal and non-optimal decision-makers (χ^2^ = 1.4, df = 1, *p* = 0.24; Figure 2B). These results suggest that neither motor impulsivity nor risk-related impulsive choice predict the acquisition of cocaine SA.

Then, the maintenance of cocaine SA was evaluated during the 15 sessions of SA. During these sessions, a mixed factorial ANOVA on the number of cocaine infusions (Figure 2C) revealed a main effect of the line [*F*_(1_,_28)_ = 7.15, *p* = 0.01] and sessions [*F*_(6_,_160)_ = 8.53, *p* < 0.0001], but no line × sessions interaction [*F*_(6_,_160)_ = 1.11, *p* = 0.36], indicating that both RHA and RLA rats increased their number of infusions over the sessions, and that RHA rats received more infusions than RLA animals throughout all sessions. However, no effect of the line [*F*_(1_,_28)_ = 2.70, *p* = 0.11], sessions [*F*_(5_,_154)_ = 1.67, *p* = 0.14], and no line by session interaction [*F*_(5_,_154)_ = 0.94, *p* = 0.46] were observed when comparing the total number of active responses between lines (Figure 2E), indicating that all rats displayed similar levels of drug seeking during the 15 sessions of cocaine SA. When comparing optimal and non-optimal rats (Figure 2D), a main effect of sessions was observed on the number of cocaine infusions, [*F*_(6_,_161)_ = 6.49, *p* < 0.0001], but no effect of profile [*F*_(1_,_28)_ = 0.0008, *p* = 0.98], nor profile × sessions [*F*_(6_,_161)_ = 0.54, *p* = 0.77], indicating no difference between optimal and non-optimal animals during the maintenance of cocaine SA. Moreover, the total number of active or inactive responses did not differ between optimal and non-optimal animals [profile: *F*_(1_,_28)_ = 0.14, *p* = 0.71; sessions: *F*_(6_,_155)_ = 1.21, *p* = 0.31; sessions × profile: *F*_(6_,_155)_ = 0.53, *p* = 0.77; Figure 2F]. These results suggest that risk-related impulsive choice is not predictive of cocaine use or cocaine seeking during maintenance of cocaine SA.

**FIGURE 3:**
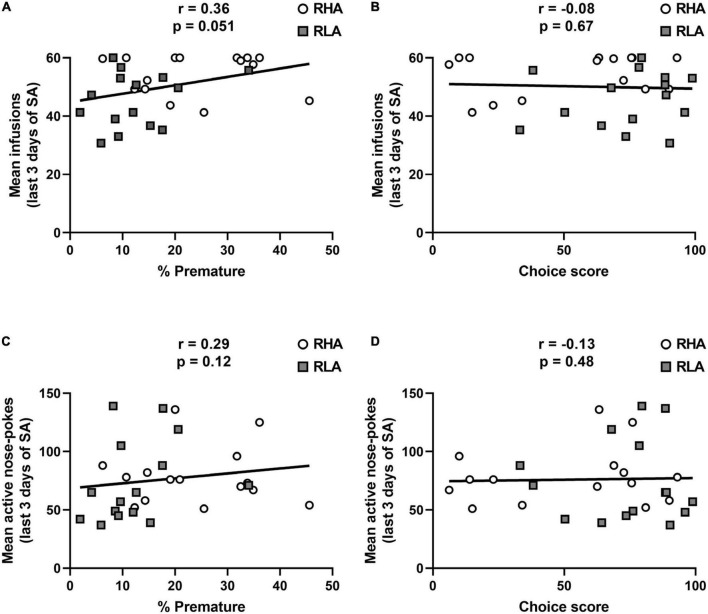
Relationships between impulsive action, risk-related impulsive choice and maintenance of cocaine SA. Correlations between the average number of infusions over the 3 last days of cocaine SA and the percentage of premature responses **(A)** and the choice score **(B)**. Correlations between the average number of total active responses over the 3 last days of cocaine SA and the percentage of premature responses **(C)** and the choice score **(D)**. *n* = 15 RHA rats; *n* = 15 RLA rats.

Irrespective of the line or decision-making profile, premature responding tended to be correlated with the average number of cocaine infusions over the 3 last days of cocaine SA (*r* = 0.36; *p* = 0.051; Figure 3A), but not with the total number of active responses (*r* = 0.29; *p* = 0.12; Figure 3C). No correlation could be detected between the choice score and neither the average number of infusions during the 3 last days of cocaine SA (*r* = −0.08; *p* = 0.67; Figure 3B), nor the total number of active responses (*r* = −0.13, *p* = 0.48; Figure 3D). Furthermore, when data were analyzed by separating RHA and RLA rats, there was no within-line correlations between the percentage of premature responses or the choice score and the number of infusions or the total number of active nose-pokes averaged over the 3 last days of cocaine SA (data not shown). These data suggest that, compared to risk-related impulsive choice, only high motor impulsivity predicts drug intake during the maintenance of cocaine SA.

### 3.3. Motivation for cocaine

**FIGURE 4:**
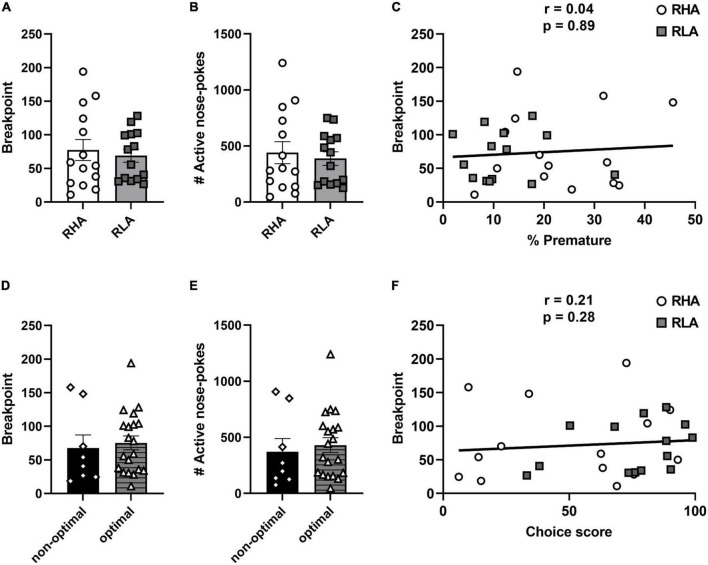
Behavioral differences in motivation for cocaine. Differences between RHA (*n* = 14) and RLA rats (*n* = 14) in BP **(A)**, and in the total number of active responses **(B)** during PR. Correlation between the BP and the percentage of premature responses **(C)**. Differences between optimal (*n* = 20) and non-optimal rats (*n* = 8) in BP **(D)**, and in the total number of active responses **(E)** during PR. Correlations between the breakpoint and the choice score in the rGT **(F)**. Data are represented as mean (± SEM) of the average of 3 PR sessions for each rat.

Rats were then tested for their motivation to consume cocaine using a PR schedule of reinforcement. No difference in BP [*t*_(26)_ = 0.45 *p* = 0.66; Figure 4A] or in the total number of active responses [*t*_(26)_ = 0.46 *p* = 0.65; Figure 4B] were found between RHA and RLA rats. Similarly, no difference in BP [t_(26)_ = 0.38 *p* = 0.71; Figure 4D] or in the total number of active responses [*t*_(26)_ = 0.45 *p* = 0.65; Figure 4E] were found when rats were categorized according to their risk-related impulsive choice profile. Further, no correlation was found between BP and the level of premature responding (*r* = 0.04; *p* = 0.86; Figure 4C) as well as between BP and the choice score (*r* = 0.21; *p* = 0.28; Figure 4F). Taken together, these data indicate that neither motor impulsivity nor risk-related impulsive choice predict the motivation for cocaine consumption.

### 3.4. Extinction and reinstatement of cocaine seeking

**FIGURE 5:**
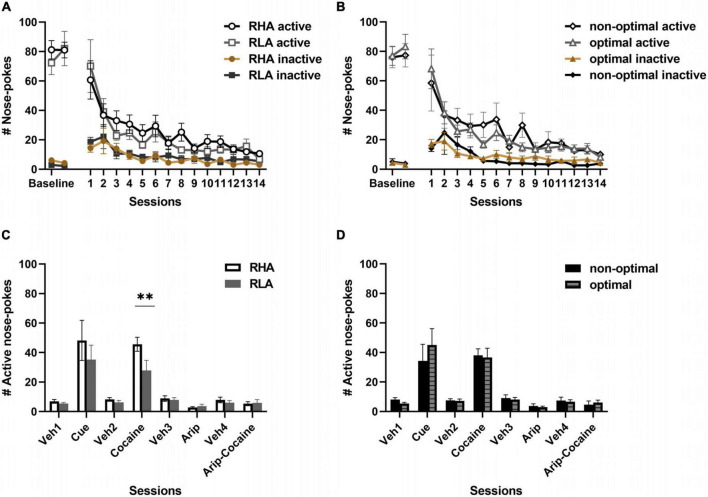
Behavioral differences in extinction and reinstatement of drug seeking behavior. Differences between RHA (*n* = 14) and RLA (*n* = 14) rats **(A)** and between optimal (*n* = 20) and non-optimal (*n* = 8) rats **(B)** in the nose-pokes decay during extinction of cocaine-seeking following discontinuation of the drug. Differences in active responses following presentation of the drug-paired cue, a priming injection of cocaine, aripiprazole or both aripiprazole and cocaine, between RHA and RLA rats **(C)** or between optimal and non-optimal rats **(D)**. Data are presented as the mean ± SEM of total active responses. **p < 0.01 RHA rats vs. RLA rats.

During the 14 days of extinction, all rats extinguished their drug-seeking behavior at a similar rate. We observed no differences in the number of total active nose-pokes between RHA and RLA rats [line: *F*_(1_,_26)_ = 0.31, *p* = 0.58; sessions: *F*_(3_,_70)_ = 15.96, *p* = 0.0001; sessions × line: *F*_(3_,_70)_ = 0.65, *p* = 0.81; Figure 5A] or between optimal and non-optimal decision-makers [profile: *F*_(1_,_26)_ = 0.19, *p* = 0.66; sessions: *F*_(3_,_69)_ = 12.21, *p* = 0.0001; sessions × profile: *F*_(3_,_69)_ = 0.71, *p* = 0.76; Figure 5B]. This suggests that neither motor impulsivity nor risk-related impulsive choice predict the extinction of drug-seeking behavior.

We then evaluated reinstatement of drug seeking induced by the drug-paired cue or the drug itself. We also assessed the effect of aripiprazole on drug-induced reinstatement of drug seeking. In RHA and RLA rats (Figure 5C), a mixed factorial ANOVA revealed a main effect of the cue [treatment *F*_(1_,_24)_ = 29.13, *p* < 0.001], but no main effect of the line [*F*_(1_,_24)_ = 1.42, *p* = 0.25] and no treatment × line interaction [*F*_(1_,_24)_ = 0.98, *p* = 0.33], indicating a similar increase in drug-seeking in both lines following presentation of the drug-paired cue. There was a main effect of cocaine priming on reinstatement of drug-seeking behavior [*F*_(1_,_23)_ = 127, *p* < 0.001], as well as a main effect of line [*F*_(1_,_23)_ = 5.77, *p* = 0.025] and an interaction between cocaine and line [*F*_(1_,_23)_ = 4.56, *p* = 0.044]. *Post hoc* comparison revealed that, although both RHA and RLA rats increased drug-seeking after a priming injection of cocaine, RHA rats reinstated more in response to the drug when compared to RLA rats (*p* = 0.006). We observed a main effect of aripiprazole [*F*_(1_,_23)_ = 16.6, *p* < 0.001] on nose-poking when compared to the vehicle session, but no effect of the line [*F*_(1_,_23)_ = 0.18 *p* = 0.67], and no interaction between treatment and line [*F*_(1_,_23)_ = 0.03, *p* = 0.85], indicating that both lines similarly decreased nose-poking in response to aripiprazole. Conversely, co-injection of aripiprazole and cocaine before session had no effect on drug seeking [treatment: *F*_(1_,_20)_ = 0.86, *p* = 0.36; line: *F*_(1_,_20)_ = 0.77, *p* = 0.39; treatment × line *F*_(1_,_20)_ = 0.04, *p* = 0.85], indicating that aripiprazole similarly prevented cocaine-induced reinstatement of drug-seeking in both high impulsive RHA rats and low impulsive RLA rats.

When comparing optimal and non-optimal decision-makers (Figure 5D), a mixed factorial ANOVA revealed a main effect of the cue, independently of the decision-making profile [treatment: *F*_(1_,_24)_ = 21.4 *p* = 0.0001; profile *F*_(1_,_24)_ = 0.009 *p* = 0.93; treatment × profile: *F*_(1_,_24)_ = 0.73 *p* = 0.40], indicating a similar increase in drug-seeking in the two groups. Additionally, a main effect of cocaine was observed [*F*_(1_,_23)_ = 95.96 *p* = 0.0001], but no main effect of the profile [*F*_(1_,_23)_ = 0.36 *p* = 0.55] nor treatment × profile interaction [*F*_(1_,_23)_ = 0.05 *p* = 0.82], as revealed by an increased number of responses in both groups. After a priming injection of aripiprazole, we observed a main effect of treatment [*F*_(1_,_23)_ = 15.25 *p* < 0.001], but no effect of group [*F*_(1_,_23)_ = 0.29 *p* = 0.60] or treatment by profile interaction [*F*_(1_,_23)_ = 0.06 *p* = 0.80], indicating that both groups similarly decreased nose-poking. No main effects were observed after co-administration of aripiprazole and cocaine [treatment: *F*_(1_,_20)_ = 1.2, *p* = 0.28, line: *F*_(1_,_20)_ = 0.003 *p* = 0.99, treatment × profile: *F*_(1_,_20)_ = 0.38, *p* = 0.54], indicating that aripiprazole blocked cocaine-induced reinstatement independently of the decision-making profile. These data suggest that risk-related impulsive choice is not predicting reinstatement of drug-seeking.

## 4. Discussion

To our knowledge, this is the first longitudinal study to investigate the predictive value of both motor impulsivity and risk-related impulsive choice on different characteristics of drug abuse. Although these two facets of impulsivity were positively correlated, and might therefore be underpinned by overlapping neurobiological mechanisms, they were differentially associated to aspects of drug abuse. We found that motor impulsivity, but not risk-related impulsive choice, predicted higher levels of cocaine intake as well as higher levels of cocaine-primed reinstatement of drug-seeking. Conversely, neither motor nor choice impulsivity were predictive of acquisition of cocaine SA, motivation to drug use, extinction of drug-seeking or cue-induced reinstatement of drug-seeking behaviors. These data indicate that high motor impulsivity may be a predisposing factor to drug abuse, while risk-related impulsive choice might be only marginally implicated in the susceptibility to abuse drugs.

In our study, motor impulsivity was not predicting the acquisition of cocaine SA, but rather the level of cocaine intake during the maintenance phase of cocaine SA. These results are in agreement with a large body of work showing that acquisition of alcohol, nicotine, or cocaine self-administration is similar between high and low motor impulsive animals (Dalley et al., 2007; Belin et al., 2008; Ferland and Winstanley, 2017; Pattij et al., 2020). Moreover, consistent with previous studies (Fattore et al., 2009; Dimiziani et al., 2019), RHA rats, which are more motor impulsive than RLAs, self-administered more cocaine than RLAs. Arguably though, the short access schedule of reinforcement used here does not capture the full complexity of psychostimulant addiction features, such as compulsive drug-seeking. Yet, our data are consistent with a previous study by Dalley et al. (2007) showing higher levels of cocaine taking in high impulsive compared to low impulsive rats, using a long and intermittent access procedure to cocaine SA. Together, these results suggest that a schedule of reinforcement using short-term access to the drug may be sufficient to study the early stages of drug abuse such as acquisition and maintenance of drug taking. On the other hand, other studies using either long (Caprioli et al., 2013), short (Abbott et al., 2022), or intermittent access (Belin et al., 2008) to cocaine SA reported similar levels of drug intake in high and low motor impulsive animals. The reason for these discrepancies is unclear but does not seem to lie in the experimental design of the schedule and duration of access to the drug.

Only a few studies have evaluated the predictive value of risk-related impulsive choice on different phases of cocaine SA and relapse (Ferland and Winstanley, 2017; Orsini et al., 2020; Hynes et al., 2021). In our study, risk-related impulsive choice was not predictive of the acquisition or the maintenance of cocaine SA, which is in line with previous work showing no differences in drug intake between optimal and non-optimal rats (Ferland and Winstanley, 2017; Orsini et al., 2020). Together, these results indicate that risk-related impulsive choice is not predictive of drug intake. Interestingly, it has been suggested that the amount of drug intake is not predictive of the development of addiction-like behaviors. Indeed, among animals taking similar amount of drugs, such as alcohol or cocaine, only a subset of animals actually develop compulsive-like behavior, a hallmark of addiction (Deroche-Gamonet et al., 2004; Giuliano et al., 2019; Goutaudier et al., 2023). Collectively with our data, these results suggest that, although risk-related impulsive choice is not predicting the levels of drug intake, it might still be predictive of other aspects of addiction-like behaviors. Thus, further studies are required to evaluate the predictive value of risk-related impulsive choice on compulsive drug use, as evaluated by a resistance to punishment (e.g., footshock) of cocaine use (Deroche-Gamonet et al., 2004).

Independently of the levels of motor impulsivity, all rats were similarly motivated for cocaine-taking as assessed by a PR schedule of reinforcement. These data substantiate previous observations showing that motivation for cocaine and alcohol is independent of the levels of motor impulsivity (Belin et al., 2008; Pattij et al., 2020). Noticeably, motivation for drug-taking has been shown to depend on the duration of drug exposure Paterson and Markou (2003), as cocaine SA under long access conditions (6 h daily) induces higher motivation for the drug than under a short access conditions (1 h daily). Thus, motivation for a drug might be independent of motor impulsivity, but rather depend on the duration of drug exposure. Interestingly, a number of studies have shown a link between motor impulsivity and “sign-tracking” (ST), a behavioral profile that is characterized by a propensity to assign incentive motivational value to reward-associated cues as opposed to “goal-tracking” (GT), a behavioral profile that is instead characterized by a propensity to assign predictive value to reward-associated cues (review in Colaizzi et al., 2020). Sign-tracking often leads to maladaptive behavior, such as the escalation of reward-seeking, and has been proposed as a model to study vulnerability to drug abuse (reviews in Sarter and Phillips, 2018; Anselme and Robinson, 2020). Similar to what is observed here and in earlier work in RHA relative to RLA rats (Giorgi et al., 1997; Tournier et al., 2013; Dimiziani et al., 2019; Bellés et al., 2023), ST animals display higher levels of motor impulsivity (Lovic et al., 2011; Swintosky et al., 2021), as well as higher risk-preference (Swintosky et al., 2021), greater locomotor sensitization following repeated cocaine exposure (Flagel et al., 2008) and stronger drug-primed reinstatement of drug-seeking (Saunders and Robinson, 2011) when compared to GT animals. Importantly, and as observed here in RHA relative to RLA rats, ST and GT animals also displayed similar motivation for drug use (Pohoøalá et al., 2021) despite their higher propensity to drug use and to drug relapse (review in Anselme and Robinson, 2020). Collectively, this suggest that a higher vulnerability to drug abuse is not only related to a higher motivation for the drug but may also proceed from other factors, such as a higher reinforcing effect of the drug in vulnerable individuals. Besides, it would be interesting in future studies to determine the ST or GT profile of RHA and RLA rats, and whether this profile is associated to their different susceptibility to drug abuse.

To our knowledge, our study is the first to evaluate the predictive value of risk-related impulsive choice on motivation for cocaine-taking. As observed for motor impulsivity, risk-related impulsive choice did not predict motivation for drug use. Interestingly, impulsive choice as assessed by delay-related decision making has also been shown not to predict motivation for alcohol or cocaine (Anker et al., 2009; Broos et al., 2012; Diergaarde et al., 2012), although high delay-discounting predicted faster acquisition of drug SA (Perry et al., 2008), higher resistance to extinction of drug seeking behavior (Diergaarde et al., 2008) and stronger cue-induced reinstatement of alcohol seeking (Pattij et al., 2020). Combined with our results, these observations suggest that, even though each facet of impulsivity might predict different aspects of drug abuse, a higher motivation for drug intake seems unrelated to impulsive behaviors.

Roman High Avoidance and RLA rats did not differ in their resistance to extinction of drug-seeking behavior, a finding consistent with a recent study showing that extinction of alcohol-seeking is similar between high and low impulsive animals (Pattij et al., 2020). However, it contrasts with previous work reporting that RHA rats, which display higher levels of motor impulsivity than RLA rats, extinguished drug-seeking behavior slower than RLAs (Fattore et al., 2009; Dimiziani et al., 2019). Although these results may appear conflicting, it should be noted that, in our study, animals were first trained on the rGT prior to cocaine SA. This major difference may have exposed rats from the present study to uncertainty, as each option in the rGT was associated with the possibility to obtain a reward or a time-out punishment. In line with this hypothesis, exposure to uncertainty has been shown to enhance amphetamine SA in drug-naïve animals (Mascia et al., 2020), suggesting that uncertainty may affect the reinforcing effects of drugs. Thus, and although not tested, it is possible that exposure to uncertainty also affected extinction of drug-seeking, and accounted, at least in part, for the lack of difference in drug-seeking under extinction conditions observed here between RHA and RLA rats. Further studies are necessary to investigate the possible consequences of exposure to uncertainty on extinction of drug-seeking behavior. Similar to what was observed when comparing RHA and RLA rats, optimal and non-optimal animals extinguished drug-seeking at a comparable level. Surprisingly, to our knowledge, only one study has evaluated extinction of drug-seeking behavior in relation to risky decision-making (Cocker et al., 2020). Congruent with our results, baseline levels of risk-related impulsive choice appeared to be unrelated to the level of responding during relapse under extinction condition (Cocker et al., 2020). On the other hand, the authors also showed that cocaine exposure led to a worsening of risk-related impulsive choice and that this worsening was predictive of relapse. This latter study, along with our own, suggests that the increased levels of risk-related impulsive choice observed in patients with SUD (Bechara et al., 2002) may likely result from drug abuse rather than predict the vulnerability to drug abuse.

We confirmed our previous work of higher cocaine-primed reinstatement of drug-seeking in RHAs compared to RLA rats (Dimiziani et al., 2019), and further showed that cue-induced reinstatement of drug-seeking was, in contrast, similar between RHA and RLA rats, but also between optimal and non-optimal rats. Previous findings, although scarce, have reported that cue-induced reinstatement of alcohol-seeking is also independent of motor impulsivity (Pattij et al., 2020). One potential explanation for the higher propensity of RHA vs. RLA rats to relapse to the drug but not to the drug-paired cue is that both lines differ in their DA response to psychostimulants, with RHA rats exhibiting a heightened release of striatal DA in response to amphetamine (Tournier et al., 2013; Bellés et al., 2021b) and cocaine (Giorgi et al., 1997) when compared to RLA rats. Thus, cocaine-priming in our study likely induced a higher striatal DA response, and consequently higher drug-seeking reinstatement, in RHAs relative to RLA rats. On the other hand, although the presentation of drug-paired cues promotes DA release (Weiss et al., 2000), the magnitude of cue-induced DA release is lower than that induced by cocaine (Weiss et al., 2000; Navailles et al., 2008) and likely leads to a reduced reinforcing effect of drug-paired cues relative to the drug itself. Together with our results, these data suggest that greater motor impulsivity, as observed in RHA vs. RLA rats, although associated to higher susceptibility to relapse, might predict specific vulnerability to drug-primed, but not cue-induced relapse.

The DA stabilizer aripiprazole has been shown to reduce cue- and cocaine-induced drug-seeking behavior in rodents (Feltenstein et al., 2007, 2009). Here, we hypothesized that aripiprazole may have a differential effect on drug-seeking in RHA and RLA rats, as the two lines differ in presynaptic DA signaling as measured by evoked DA release (Tournier et al., 2013; Bellés et al., 2021b). However, here, aripiprazole by itself reduced nose-poke responding when compared to vehicle conditions, independently of the levels of motor impulsivity. These results are in line with previous studies showing that aripiprazole reduced impulsive behaviors similarly in high- and low-impulsive animals (Besson et al., 2010; Bellés et al., 2023). However, we cannot exclude the possible contribution of an aripiprazole-induced change in locomotor activity in this effect. At the dose used here (1 mg/kg), mixed results exist on the effect of aripiprazole on locomotion, with data showing a reduction (Feltenstein et al., 2007), and others no effect (Viana et al., 2013) on locomotor activity. Notwithstanding this, our results suggest that, despite its partial agonistic D_2/3_R properties, aripiprazole is devoid of reinforcing properties, as previously suggested in a study showing that rodents failed to self-administer, and even reduced nose-poking behavior when self-administering aripiprazole (Sørensen et al., 2008). Additionally, aripiprazole blocked cocaine-primed reinstatement of drug-seeking independently of the line or decision-making profile. Although cocaine-induced DA release is higher in RHAs than in RLA rats (Giorgi et al., 1997; Tournier et al., 2013), cocaine is known to induce massive striatal DA release (Navailles et al., 2008). Then, it is possible that the high magnitude of cocaine-evoked DA release exceeded the intrinsic partial agonist activity of aripiprazole. As such, aripiprazole likely displayed antagonistic properties in both lines, thus leading to a similar blockade of cocaine-primed reinstatement of drug-seeking behavior (Feltenstein et al., 2007, 2009). In line with this hypothesis, previous observations reported that aripiprazole pretreatment prevents cocaine SA independently of the dose of cocaine used (Sørensen et al., 2008). Another explanation for the similar effect of aripiprazole on cocaine-primed reinstatement of drug-seeking in RHA and RLA rats might be its action on serotoninergic (5HT) receptors. Indeed, although aripiprazole has a higher affinity for D_2/3_R, it also displays antagonistic properties at the 5HT_2*a*_R (Natesan et al., 2006; Casey and Canal, 2017). Similar to what has been observed using D_2/3_R antagonists (Anderson et al., 2006), 5HT_2*a*_R antagonism reduces cocaine-primed reinstatement of drug-seeking (Fletcher, 2002). Interestingly, compared to RLA rats, RHAs display higher levels of 5HT_2*a*_R binding associated to their level of impulsivity (Klein et al., 2014). Thus, the effects of aripiprazole on the blockade of cocaine-primed reinstatement, as observed in our study may result from its combined action on 5HT_2*a*_R and D_2/3_R. Collectively, these data confirm that aripiprazole is devoid of reinforcing properties and might be an effective therapeutic agent to prevent cocaine-primed relapse, and further showed that this effect is independent of inter-individual differences in basal level of impulsivity.

We further strengthened here the existence of a positive association between motor impulsivity and risk-related impulsive choice, as already reported (Barrus et al., 2015; Ferland and Winstanley, 2017; Cho et al., 2018; Bellés et al., 2023). Such an association supports clinical observations showing higher levels of motor impulsivity in pathological gamblers (Chowdhury et al., 2017), individuals who are more prone to take risky decisions in the Iowa Gambling Task (reviewed in Brevers et al., 2013). Furthermore, motor impulsivity has been related to a decreased thickness of the prefrontal cortex in humans, suggesting a reduced cortical activity in high impulsive individuals (Lim et al., 2021). Interestingly, lesions of the frontal cortex in humans is also associated with riskier decision making (Bechara et al., 1994). Similarly, in rodents, pharmacological inactivation or lesioning of the medial prefrontal cortex (mPFC) increased motor impulsivity (Feja and Koch, 2014), and risk-related impulsive choice in rats (Paine et al., 2013; Zeeb et al., 2015). Previous studies indicated that RHA rats display a lower gray matter volume in the mPFC when compared to RLA rats (Río-Álamos et al., 2019), thus suggesting that a lower cortical activity might be responsible for the higher motor impulsivity and riskier decision-making observed in RHA rats. Noticeably, mPFC neurons project on the nucleus accumbens (Takagishi and Chiba, 1991; Vertes, 2004), a brain structure involved in both motor impulsivity and risk-related impulsive choice (review in Basar et al., 2010). Further studies investigating the effects of modulating the activity of mPFC-projecting neurons to the nucleus accumbens on impulsive action and risk-related impulsive choice would be interesting. Such studies could help to define the underlying neural circuits involved in the different facets of impulsivity.

In the present study, only male rats were tested. However, there is an increased body of evidence showing sex differences in impulsivity and vulnerability to drug abuse. In rodents, females acquire drug SA faster, take more drug, are more motivated for drug taking, and reinstate drug-seeking more than male animals (review in Becker, 2016). On the other hand, and despite a higher propensity to drug abuse in females, current evidence also indicate that females display lower levels of motor impulsivity (Bayless et al., 2012), and are more risk-avoidant (Ishii et al., 2018; Orsini et al., 2020) than males. Although it may seem contradictory to the hypothesis that impulsivity is a predictor for drug abuse, it is important to note that impulsivity is not the only candidate risk factor for drug abuse. Other factors, such as novelty-seeking and anxiety, have also been associated to a higher propensity to drug abuse (review in Belin et al., 2016). Females have been shown to exhibit higher levels of anxiety-related behaviors than males (Toufexis et al., 2005; Bishnoi et al., 2020). Thus in females, other risk factors, such as anxiety-related behaviors, might prevail over impulsive behaviors to predict a higher vulnerability to drug abuse when compared to males. On the other hand, male RHA rats are less anxious than their male RLA counterparts (review in Giorgi et al., 2019), and still are more vulnerable to drug abuse than RLAs. Thus, in male RHA rats, high impulsive behaviors (Bellés et al., 2023) and novelty seeking (Tournier et al., 2013; Bellés et al., 2021b) may predominate over anxiety-related behaviors to predict greater vulnerability to drug abuse. Therefore, a higher susceptibility to drug abuse may result from the conjunction and/or complex interplay of different risk-factors within an individual depending on biological variables such as sex.

## 5. Conclusion

Our study highlighted that motor impulsivity, but not risk-related impulsive choice, is predicting drug-taking and drug-primed relapse. We complement and strengthen arguments in favor of using motor impulsivity as a predictive factor for vulnerability to drug abuse. However, although we did not find any evidence for the utility of risk-related impulsive choice in predicting drug abuse, this does not rule out the involvement of this facet of impulsivity on other fundamental aspects of drug abuse that have not been explored here, such as compulsive drug use or preference for drugs over natural reward. Further studies are needed to determine whether maladaptive risk-related impulsive choice, as observed in patients with SUD (Bechara et al., 2002), is a predictive factor for vulnerability to SUD or a consequence of drug abuse.

## Data availability statement

The raw data supporting the conclusions of this article will be made available by the authors, without undue reservation.

## Ethics statement

This animal study was reviewed and approved by the Animal Ethics Committee of the Canton of Geneva.

## Author contributions

CA, GU-M, and LB acquired the data. CA analyzed the data and wrote the first draft of the manuscript. CA, RG, and NG revised the manuscript and wrote the final version of the manuscript. NG conceptualized and designed the study. All authors critically reviewed the content and approved the submitted version.
